# The Application of Nano Zero-Valent Iron in Synergy with White Rot Fungi in Environmental Pollution Control

**DOI:** 10.3390/toxics12100721

**Published:** 2024-10-02

**Authors:** Guoming Zeng, Zilong Ma, Rui Zhang, Yu He, Xuanhao Fan, Xiaoling Lei, Yong Xiao, Maolan Zhang, Da Sun

**Affiliations:** 1 School of Civil and Hydraulic Engineering, Chongqing University of Science and Technology, Chongqing 401331, China; 2 Chongqing Academy of Science and Technology, Chongqing 401123, China; 3 School of Civil Engineering, Chongqing Jiaotong University, Chongqing 400074, China; 4 Intelligent Construction Technology Application Service Center, Chongqing City Vocational College, Chongqing 402160, China; 5 School of Metallurgy and Power Engineering, Chongqing University of Science and Technology, Chongqing 401331, China; 6 National & Local Joint Engineering Research Center for Ecological Treatment Technology of Urban Water Pollution, College of Life and Environmental Science, Wenzhou University, Wenzhou 325035, China; 7 Zhejiang Provincial Key Laboratory for Water Environment and Marine Biological Resources Protection, College of Life and Environmental Science, Wenzhou University, Wenzhou 325035, China; 8 Institute of Life Sciences, Biomedical Collaborative Innovation Center of Zhejiang Province, Wenzhou University, Wenzhou 325035, China

**Keywords:** nano zero-valent iron, white rot fungi, pollutant removal mechanisms, synergistic effects

## Abstract

Developing efficient and sustainable pollution control technologies has become a research priority in the context of escalating global environmental pollution. Nano zero-valent iron (nZVI), with its high specific surface area and strong reducing power, demonstrates remarkable performance in pollutant removal. Still, its application is limited by issues such as oxidation, passivation, and particle aggregation. White rot fungi (WRF) possess a unique enzyme system that enables them to degrade a wide range of pollutants effectively, yet they face challenges such as long degradation cycles and low degradation efficiency. Despite the significant role of nZVI in pollutant remediation, most contaminated sites still rely on microbial remediation as a concurrent or ultimate treatment method to achieve remediation goals. The synergistic combination of nZVI and WRF can leverage their respective advantages, thereby enhancing pollution control efficiency. This paper reviews the mechanisms, advantages, and disadvantages of nZVI and WRF in pollution control, lists application examples, and discusses their synergistic application in pollution control, highlighting their potential in pollutant remediation and providing new insights for combined pollutant treatment. However, research on the combined use of nZVI and WRF for pollutant remediation is still relatively scarce, necessitating a deeper understanding of their synergistic potential and further exploration of their cooperative interactions.

## 1. Introduction

In the face of increasing global environmental pollution, there is a growing focus on developing efficient and sustainable technologies for remediating pollutants. Pollution removal and remediation have always been central concerns in the field of environmental science. In recent years, there has been a growing focus on using nanomaterials and microorganisms for environmental pollutant remediation. Nanomaterials have unique physicochemical properties, including large specific surface areas, abundant active sites, and adjustable surface properties, making them ideal for the adsorption and catalytic degradation of pollutants. Microorganisms, being highly metabolically capable and selective, can degrade specific pollutants under certain conditions, showing great potential for practical applications. Nanoparticles are ubiquitous and coexist with microorganisms in various environments on Earth. The interactions between microorganisms and nanoparticles influence biogeochemical cycles by accelerating reaction rates and facilitating biological processes at microscales [1]. Therefore, utilizing nanomaterials in conjunction with microorganisms for the remediation of pollutants through synergistic actions is considered to be an effective and feasible approach.

Nano zero-valent iron (nZVI) is a type of nanomaterial known for its large specific surface area, high reactivity, and strong adsorption capacity. Unlike traditional remediation materials, nZVI not only has higher removal efficiency but also requires lower dosages to effectively adsorb and remove pollutants. However, its application is limited due to potential toxicity, stability issues, and susceptibility to oxidation and passivation during long-term use. White-rot fungi (WRF) are a type of microorganism that belongs to the Basidiomycota class. They have strong capabilities for degrading various pollutants because of their unique metabolic pathways and mechanisms for breaking down large substances outside the cell. Enzymatic and free radical reactions are key processes in this degradation [2]. However, the degradation cycle of WRF for pollutants is relatively long, and their degradation efficiency is not high. Additionally, their growth cycle is lengthy, and enzyme activity is limited by various factors. Some studies have shown that combining WRF with nanomaterials can enhance their biotechnological conversion efficiency and reduce processing time [3].

This paper summarizes and reviews the research progress on nZVI and WRF in the field of pollutant remediation and explores the potential applications of their combined use. Firstly, the advantages and disadvantages, removal mechanisms, and applications of nZVI and WRF in pollutant remediation are introduced, with a focus on their application in the treatment of different types of pollutants, such as heavy metals and organic pollutants. Secondly, the advantages of combining nZVI with microorganisms and their interactions are reviewed, explaining the principles and influencing factors of their synergistic effects. Finally, the practical applications of combining nZVI with WRF for pollutant remediation are explored, emphasizing their potential in pollutant control. However, research on their combined use is relatively scarce, and future studies should focus on deeply understanding the synergistic potential of WRF and nZVI and exploring their collaborative interactions. Through an in-depth exploration of the combined remediation technology of nZVI and WRF, new insights and methods for addressing environmental pollution issues are expected to emerge, driving innovation and development in environmental remediation technologies and promoting the achievement of sustainable development goals. This paper aims to review the research progress of nZVI and WRF in the field of pollutant remediation, exploring the potential prospects of their combined application, analyzing their advantages and synergistic mechanisms in pollutant treatment, and providing valuable insights for future research on the joint application of nZVI and WRF.

## 2. Nano Zero-Valent Iron

**Figure 1:**
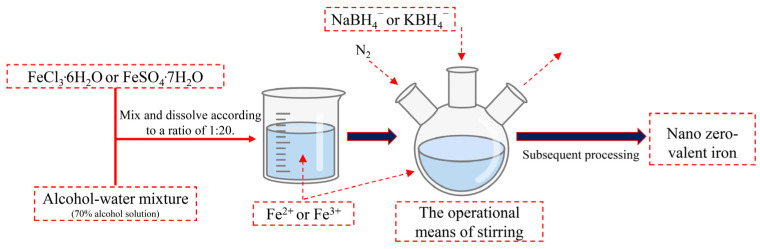
Flowchart of the liquid-phase reduction method for preparing nZVI.

nZVI stands for zero-valent iron (ZVI) particles with diameters ranging from 1 to 100 nm. These nanomaterials are known for their large specific surface area, strong reducibility, and high adsorption capacity [4,5,6]. The earliest research on nZVI can be traced back to 1995 when Glavee et al. [7] first prepared nZVI powder by borohydride reduction in Fe (II) and Fe (III). In comparison to ZVI, nZVI exhibits a larger specific surface area and higher reactivity, enabling direct injection into contaminated areas for in situ remediation. It serves as an efficient material for removing or degrading various pollutants, demonstrating promising applications in water pollution treatment systems, and is among the most extensively researched nanomaterials in the field of water treatment [8,9,10,11,12,13]. In recent years, there has been extensive exploration of preparation methods for nZVI, with liquid-phase reduction being a commonly employed technique. The fundamental principle involves using a potent reducing agent, BH4-, under the protection of inert gases like nitrogen or argon to convert Fe^2+^ or Fe^3+^ to ZVI, thus facilitating the synthesis of nZVI. In laboratory preparations, NaBH_4_ or KBH_4_ solutions are typically chosen as reducing agents, and the reaction is carried out in a liquid-phase environment. The process flow diagram for the preparation of nZVI via liquid-phase reduction is depicted in Figure 1 [14].

Green synthesis, as an alternative and environmentally friendly technique for nZVI synthesis, has gained increasing attention in recent years; this method offers advantages such as cost-effectiveness, ease of preparation, reduced chemical usage, reliability, and environmental friendliness [15]. It uses bioactive substances from plant extracts to reduce Fe (III) or Fe (II) to Fe (0). Plant extracts contain reducing agents such as polyphenols and flavonoids, which not only reduce Fe (III) or Fe (II) but also effectively prevent nanoparticle aggregation [16]. Some studies have used plant extracts to synthesize nanoscale zero-valent iron (nZVI). For example, plant extracts were prepared by boiling 60 g/L of Damascus rose, thyme, and nettle leaves at 80 °C for 1 h [17]; walnut husk extract was prepared by boiling 20 g/L of walnut husks at 60 °C for 20 min [18]; and pomegranate peel extract was made by boiling 60 g/L of pomegranate peels at 80 °C for 1 h, with the remaining peels filtered using a vacuum pump [19]. Additionally, 15 g of dried eucalyptus leaves were boiled in 250 mL of deionized water at 80 °C for 1 h, and the extract was vacuum filtered and stored at 4 °C for future use. Green tea extract was prepared with a similar process [20]. Other plants that can be used to extract bioactive reducing agents include vine leaves, black tea, and more. The green synthesis method for nZVI, using plant extracts instead of traditional chemical-reducing agents, not only avoids the use of harmful chemicals and reduces environmental pollution but also significantly lowers synthesis costs. Plant extracts are widely available, inexpensive, and rich in active compounds such as polyphenols and flavonoids, which not only effectively reduce iron ions but also prevent nanoparticle agglomeration, thereby enhancing the stability and reactivity of nZVI. In addition, the green synthesis process is simple, requires no complex equipment or techniques, and is suitable for large-scale production. With its advantages of being environmentally friendly, low-cost, highly stable, and efficient, the green synthesis method has gained increasing attention in the preparation of nZVI and shows great potential for applications in environmental remediation and other fields.

### 2.1. Mechanisms of Pollutant Removal by nZVI

The process of nZVI remediating water involves multiple sequential reactions, including adsorption, reduction, precipitation, and oxidation (in the presence of dissolved oxygen). These reactions work together to complete the overall remediation mechanism [6,21]. Due to its extremely small size and large specific surface area, nZVI exhibits excellent adsorption performance, reduction capability, and reactivity. In anaerobic conditions, based on Equations (1) and (2), Fe^0^ can be oxidized by H_2_O or H^+^, generating Fe^2+^ and H_2_, which may also act as potential reducing agents for pollutants. Therefore, due to corrosion, there are three main reducing agents in the Fe-H_2_O system: Fe, Fe^2+^, and H_2_ [22]. The removal process of organic pollutants involves two key steps: first, the oxidation of Fe^0^, followed by the reduction in organic pollutants [23]. In this process, Fe^0^ releases electrons, which are used to reduce organic pollutants. Equation (3) shows the reaction of Fe^0^ releasing electrons.

Fe + 2H_2_O→Fe^2+^ + H_2_ + 2OH^−^ (1)

Fe + 2H^+^→Fe^2+^ + H_2_ (2)

Fe^0^→Fe^2+^ + 2e^−^ (3)

The removal of pollutants involves coagulation or precipitation, where pollutants and intermediates coagulate with Fe (II) and Fe (III) oxides or hydroxides. According to Equations (4)–(6), Fe^2+^ is oxidized to Fe^3+^, and then Fe^3+^ and Fe^2+^ react with OH^−^ to form iron hydroxides, ultimately removing pollutants through coagulation or precipitation [24]. Additionally, the corrosion of Fe^0^ can lead to an increase in pH in weakly buffered systems, with the effect being more pronounced under aerobic conditions due to the faster corrosion rate. Fe^3+^ is the oxidation product of the redox reaction between Fe^2+^ and pollutants. The pH increase associated with corrosion favors the formation of Fe (OH)_3_, which is an effective flocculant [25].

Fe^2+^→Fe^3+^ + e^−^ (4)

Fe^2+^ + 2OH^−^→Fe (OH)_2_ (5)

Fe^3+^ + 3OH^−^→Fe (OH)_3_ (6)

Dissolved oxygen (DO) plays a crucial role in the degradation of pollutants by nZVI. Under aerobic conditions, the corrosion of Fe produces hydrogen peroxide (H_2_O_2_). According to Reactions (7)–(9), the presence of DO promotes the generation of hydroxyl radicals (OH), which are sufficiently strong oxidants to effectively degrade a variety of organic compounds [22]. According to Reactions (10)–(12), during the reaction process, Fe^3+^ reacts with H_2_O_2_, regenerating Fe^2+^ and∙O_2_^−^, the cyclic reactions between Fe^2+^, Fe^3+^, ∙OH, and ∙O_2_^−^ continue throughout the process, and based on Reactions (13) to (14), the conversion of ∙O_2_^−^ can produce ^1^O_2_ [26,27].

Fe^0^ + O_2_ + 2H^+^→Fe^2+^ + H_2_O_2_ (7)

Fe^0^ + H_2_O_2_ + 2H^+^→Fe^2+^ + H_2_O (8)

Fe^2+^ + H_2_O_2_→Fe^3+^ +∙OH + OH^−^ (9)

Fe^3+^ + H_2_O_2_→Fe^2+^ + 2H^+^ + O_2_^−^ (10)

Fe^2+^ +∙OH→Fe^3+^ + OH^−^ (11)

Fe^3+^ +∙O_2_^−^→Fe^2+^ +O_2_ (12)

2O_2_^−^ + H_2_O→^1^O_2_ + H_2_O_2_ + 2OH^−^ (13)

O_2_^−^ + H^+^→^1^O_2_ + H_2_O_2_ (14)

In summary, the process of water remediation by nZVI involves multiple sequential reactions, including adsorption, reduction, precipitation, and oxidation. These reactions collectively contribute to the completion of the overall remediation mechanism. nZVI effectively captures and immobilizes pollutant molecules through adsorption, thereby reducing their concentration in water. In the reduction reactions, electrons released by nZVI are used to reduce organic and inorganic pollutants, decreasing their toxicity and reactivity and facilitating the restoration of the aquatic environment. The coagulation and precipitation processes facilitate the binding of pollutants to the generated precipitates, achieving the separation and removal of the pollutants. In oxidation reactions, the presence of dissolved oxygen accelerates the generation of hydroxyl radicals, aiding in the oxidative degradation of organic compounds and further enhancing the purification effect on water quality. Integrating these reaction processes, the mechanism of pollutant removal by nZVI is multi-level and multi-faceted, achieving efficient and comprehensive pollution purification through synergistic interactions.

### 2.2. Limitations of nZVI

**Figure 2:**
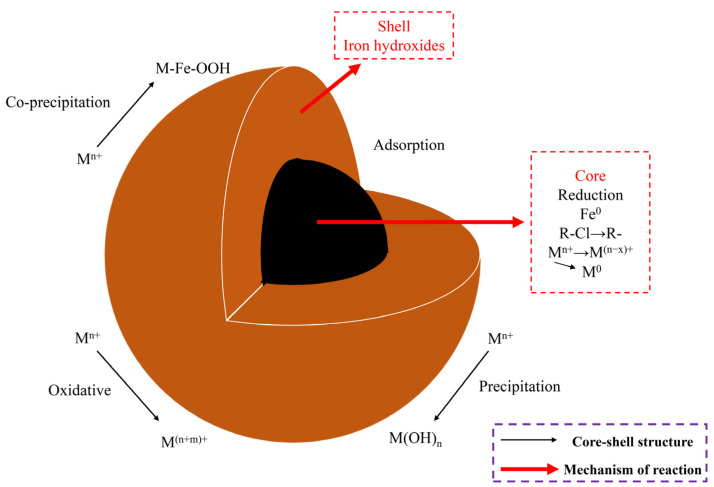
Structural model and reaction mechanisms of nZVI.

Despite its widespread application, nZVI also has certain limitations. Due to the small particle size, large specific surface area, and high surface energy of nZVI, surface effects cause the interparticle binding force to exceed its own weight, leading to a lack of stability. Additionally, the magnetic interactions between nZVI particles make them prone to agglomeration, significantly reducing the specific surface area available for reactions and thereby decreasing degradation efficiency [8,28,29]. nZVI is prone to oxidation and passivation. Brief contact with air and water can cause it to oxidize into Fe (II) or Fe (III), significantly reducing its ability to reduce other substances. If nZVI is highly pure and comes into direct contact with air, it can ignite spontaneously or produce sparks. Gradual exposure to oxygen in the air can result in the formation of an iron oxide shell on the surface, which reduces its reactivity [30,31,32]. The chemical potential gradient at the the interface between water and nZVI involves the transition of Fe species from outer Fe (III) oxides to a mixed Fe (III)/Fe (II) layer, followed by Fe (II) oxides and pure Fe (0), creating an ideal pathway for electron transfer for the reduction in surface-bound pollutants [33]. The core–shell structure of nZVI endows it with excellent overall performance. The metallic iron acts as an electron donor, providing reduction properties, while the oxide shell facilitates or inhibits electron transfer from the metallic core to the shell through electrostatic interactions and/or surface complexation. The core–shell structure is primarily composed of iron oxides, which are formed during the synthesis of nZVI through the corrosion of the iron core by water, oxygen, and substrates; as the exposure distance increases, the oxide phases sequentially form FeO, Fe_3_O_4_, and Fe_2_O_3_. The oxide shell of nZVI is initially composed mainly of conductive iron oxides (such as FeO and Fe₃O₄), and a new hydroxyl oxide shell composed of FeOOH will form with prolonged reaction time in the solution [15]. The reduction process of pollutants relies on the indirect electron transfer from the iron core through the oxide shell to the adsorbed pollutants. Generally, iron oxide is considered an n-type semiconductor, and its electrical migration is related to the energy required to excite electrons from the valence band to the conduction band (i.e., Eg); the Eg value of FeOOH is significantly higher than that of FeO and Fe₃O₄, which indicates that FeOOH will inhibit electron transfer from the iron core to the iron oxide shell, thereby slowing down the reduction in pollutants, FeOOH primarily acts as a physical barrier between the underlying metal and dissolved oxidants, suppressing electron transfer [15,34]. In the absence of modification, the majority of synthesized nZVI particles are spherical. The structural model and reaction mechanisms of nZVI are illustrated in Figure 2 [35].

### 2.3. Modification of nZVI

**Figure 3:**
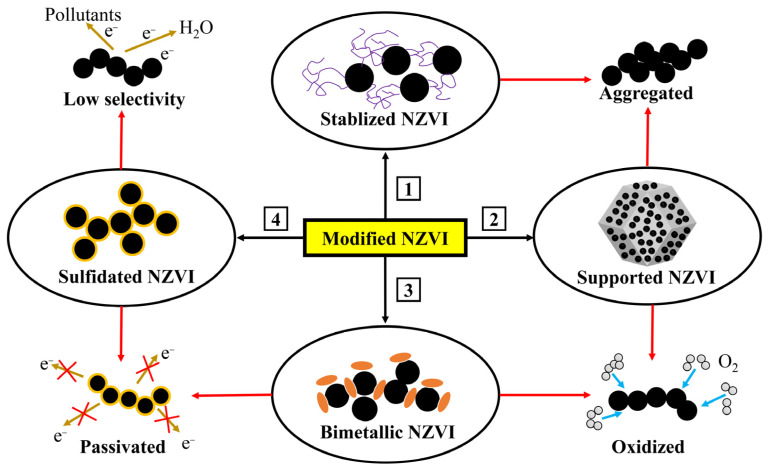
Schemes follow the same formatting. Diagram of modification strategies of nZVI (black arrows) and its application drawbacks (red arrows) overcome by modified nZVI.

Currently, many studies are focused on enhancing the stability and reactivity of nZVI through modification. These modification approaches typically include stabilized nZVI, bimetallic nZVI, supported nZVI, and sulfided nZVI (S-nZVI). The limitations of these modification strategies for nZVI and their respective applications are illustrated in Figure 3 [36].

#### 2.3.1. Sulfidized nZVI

To enhance the stability and reactivity of nZVI, S-nZVI is one of the most promising methods to inhibit the surface oxidation of nZVI and accelerate electron transfer. The sulfidation process forms an FeS shell on the nZVI surface, reducing its contact area with water and thus effectively inhibiting the oxidation of nZVI; hydrophobic iron sulfides such as tetragonal ferrosulfide and pyrite have a higher affinity for pollutants [36,37,38]. The superior performance of S-nZVI can be attributed to its unique core–shell structure, which provides exceptional adsorption and reduction capabilities [39,40]. Li et al. [41] synthesized sulfidated nZVI coated with alginate (S-nZVI@alginate) and evaluated its debromination activity towards tetrabromobisphenol A (TBBPA). Experimental results indicated that S-nZVI@alginate accelerated the removal process of TBBPA.

Sulfidation, being a widely used modification technique, is essential in preventing the oxidation and agglomeration of nZVI, speeding up electron transfer rates, and increasing the specific surface area, which collectively improves the efficiency of pollutant removal in water. However, although sulfidation can enhance the stability of nZVI, its ability to improve oxidation resistance is limited, and the rapid electron transfer rate can lead to the reaction of S-nZVI with water to produce hydrogen gas, thereby reducing the removal efficiency. Therefore, it is necessary to combine sulfuration with other modification methods to explore more effective functionalization strategies for preparing more efficient materials. Sulfidation enhances the hydrophobicity of the nZVI surface, decreasing the nano-iron’s reduction capacity towards water and thus reducing the ineffective loss of electrons. Currently, the research on pollutant removal by S-nZVI mainly involves three mechanisms: adsorption, oxidation, and reduction. These studies provide important references for a deeper understanding of the mechanisms of S-nZVI in environmental remediation and point to directions for future research and applications.

#### 2.3.2. Supported nZVI

Supported nZVI not only increases the specific surface area of the material, enhancing its contact area with pollutants, but also accelerates the transfer of pollutants to active sites through the adsorption properties of the support itself, effectively preventing nZVI agglomeration [36,42,43,44]. Zhou et al. [45] successfully synthesized chitosan-coated nano-iron-nickel bimetallic particles (CS-Fe-Ni), which enhanced the dispersion stability and mobility of the nano-iron. Kustov et al. [46] attempted to use three types of modified chitosan-stabilized Pd-nZVI nanoparticles to degrade the perchloromethane (PCE), successfully degrading 0.05 mmol of PCE within 180 min. Xue et al. [47] synthesized the composite material of nanosized zero-valent iron and garlic biomass (CMGP-nZVI) using a liquid-phase reduction method for the degradation and purification of methylene blue in aqueous solutions. The results indicated that CMGP-nZVI exhibited significantly better performance in treating methylene blue than garlic waste alone, with the loading of nZVI markedly enhancing the adsorption capacity. Cheng et al. [48] investigated the phosphorus removal efficiency of porous ceramsite loaded with nZVI. The study revealed that the composite material not only increased the dispersion and stability of the nZVI particles but also significantly improved the treatment efficacy of phosphorus-containing wastewater due to the synergistic adsorption properties of the porous material. Shi et al. [49] successfully prepared bentonite supported nano zero-valent iron (B-nZVI) using the sodium borohydride reduction method and applied it for the removal of Cr (VI) from water. In the supported nZVI system, bentonite has been demonstrated to act as an effective dispersant and stabilizer, effectively reducing the agglomeration of Fe^0^ and thereby increasing the removal efficiency of Cr (VI). The study results indicated that, compared to plain nZVI, B-nZVI exhibited superior removal efficiency, with removal rates of 100% and 60%, respectively.

In conclusion, supported nZVI can effectively address the drawbacks of nZVI, such as its tendency to agglomerate and its low reactivity. This enhancement increases the contact area between the nanoparticles and pollutants. Additionally, the use of supported nZVI can prevent the release of large quantities of nanoparticles into the environment, reducing potential environmental hazards and improving pollutant removal efficiency and stability. Overall, this demonstrates a promising prospect for application.

#### 2.3.3. Varying Reactivity of nZVI

The nZVI synthesized through various methods exhibits differences in structural configuration, particle size distribution, and surface area. These distinctions influence nZVI’s reactivity and aggregation behavior, potentially resulting in diverse outcomes in remediation efficiency and toxicity. Gil-Díaz et al. [50] compared the effectiveness of three commercial ZVI nanoparticles in stabilizing arsenic and mercury in two soil types. At lower doses of nZVI, the stabilization effect on arsenic was significant, whereas mercury required higher doses to effectively reduce its exchangeable content. The stabilization effectiveness of metals (metalloids) depends on the type of nZVI, soil properties, and contaminant characteristics. Different nZVIs exhibit varying effects in stabilizing arsenic and mercury, with nano-iron being more effective for arsenic, while RNIP and RNIP-D perform better for mercury. The remediation efficiency of commercial nZVI varies depending on the metal (metalloid), necessitating further laboratory-scale experiments to determine the most suitable nZVI type for each contaminated site. Eljamal et al. [51] used four different stabilized nZVIs, including polyacrylamide nZVI (PAA-nZVI), carboxymethyl cellulose nZVI (CMC-nZVI), polyethylene sorbitan monolaurate nZVI (PSM-nZVI), and polyvinylpyrrolidone nZVI (PVP-nZVI), as well as bare nZVI, to compare the highest nitrate and phosphate removal rates. The study found that the nitrate reduction efficiency was as follows: PVP-nZVI > PAA-nZVI > PSM-nZVI > CMC-nZVI > bare nZVI. The phosphate removal rates were as follows: PAA-nZVI > bare nZVI > PSM-nZVI > PVP-nZVI > CMC-nZVI. The results indicated that PAA-nZVI demonstrated superior performance in removing nitrate and phosphate across a broad pH range compared to other nZVIs. Mokete et al. [52] studied the reactivity of three metal-doped nZVIs (bimetallic), including Fe-Cu, Fe-Ni, Fe-Ag bimetallic particles, and nZVI in aqueous solution. nZVI and its bimetallic nanoparticles underwent oxidation during the reaction. nZVI showed the highest reactivity under acidic conditions, followed by FeNi, FeAg, and FeCu, with bimetals showing slightly lower activity due to the shielding effect on the iron core. Under oxygen-rich conditions, FeNi exhibited the best short-term performance within 3 h. The nitrate removal efficiency under alkaline conditions was in the order of FeCu, FeAg, FeNi, and nZVI. The effect of temperature on the different bimetals varied: FeCu performed best at 90 °C, FeNi had the highest iron dissolution at both 5 °C and 90 °C, while FeAg was most reactive at 25 °C. Doping with Ni, Cu, and Ag increased surface area, delayed oxidation, and enhanced electron transfer ability. The reactivity of nZVI and bimetals differed significantly under varying conditions, with FeNi showing excellent short-term performance, FeCu being suitable for high temperatures, and FeAg performing well in moderate-temperature environments.

The selection and optimization of nZVI types should be based on the characteristics of the contaminants and the conditions of the soil or water, in combination with laboratory-scale tests to determine the most suitable type of nZVI. Simultaneously, in-depth exploration of the effects of different preparation methods on the long-term stability and reactivity of nZVI is essential. By utilizing diversified stabilizers and metal doping strategies, the performance of nZVI under complex environmental conditions can be further enhanced, improving its remediation effectiveness for various pollutants to achieve broader practical applications. Through detailed research and optimization of nZVI’s varying reactivities, the application in pollution control can become more widespread and efficient.

## 3. nZVI Combined with Microorganisms

Although nZVI plays a significant role in pollutant remediation, most contaminated sites still rely on bioremediation as a concurrent or ultimate treatment approach to achieve remediation goals. Microbial communities are not only crucial for nutrient cycling in ecosystems but also play a key role in the degradation and immobilization of pollutants and heavy metals [53]. Bioremediation is an efficient, environmentally friendly, and cost-effective approach. Since it is a natural process, it results in minimal adverse effects on the environment [54,55,56]. However, bioremediation is influenced by various environmental factors, such as changes in oxygen levels, pH, and ambient temperature, all of which can affect its effectiveness. Additionally, due to the limitations of biological metabolic efficiency, the rate of bioremediation is typically slow, resulting in relatively long degradation cycles for pollutants, making it difficult to observe significant changes in the short term. Consequently, in recent years, the integration of bioremediation with other technologies has emerged as a primary research direction. Combined remediation technology involves integrating two or more methods to overcome the limitations of individual approaches and enhance remediation efficiency, thereby reducing costs and improving effectiveness. Consequently, some research has focused on integrating nZVI with microorganisms to exploit their synergistic benefits for pollution remediation.

### 3.1. The Impact of nZVI on Microorganisms

The corrosion of nZVI can potentially decrease the oxidation–reduction potential (ORP), thus establishing an appropriate reducing environment conducive to the growth of anaerobic bacteria [57]; the hydrogen gas generated at the cathode during the corrosion of nZVI serves as a preferred electron donor for various microorganisms, including *dehalogenating bacteria (DHB)*, methanogenic, *denitrifying bacteria (DNB)*, and *sulfate-reducing bacteria (SRB)* [58,59,60]. The iron oxides and hydroxides generated from nZVI corrosion can improve the biodegradation efficiency of *iron-reducing bacteria (IRB)* by supplying electron acceptors and decreasing the toxicity of pollutants to the bacteria [57]. Certain *iron-oxidizing bacteria (IOB)* are capable of using H^+^ directly from the nanoparticles that form on their cell walls [1,53]. Additionally, microbial cells can avert self-toxicity by secreting metal-binding compounds into the extracellular environment, where these metals are chelated, thus preventing their entry into the cells via non-specific membrane transport proteins [61]. In addition, iron precipitation assists in preventing the buildup of toxic metabolic byproducts (such as H_2_S) and also hinders the penetration of nanoparticles into cells, thus promoting microbial survival or encouraging the formation of biofilms [1,62,63]. Furthermore, studies measuring dehydrogenase activity show that lower concentrations of nZVI (20 and 50 mg/L) can enhance microbial activity. Moreover, nZVI does not have any harmful effects on the total bacterial abundance in the microenvironment, and nZVI with a biodegradable polyaspartic acid coating can increase bacterial numbers [64,65]. The corrosion and dissolution of nZVI not only serve as a source of trace elements and electron donors (such as H_2_/[H]) but also enhance the system’s buffering capacity and adjust the oxidation–reduction potential, thereby creating a favorable thermodynamic environment. This environment supports the growth of *organic-degrading bacteria (ODB)* and *hydrogenotrophic methanogens (HM)*, consequently enhancing the relative abundance of microorganisms [66]. nZVI is highly reactive to oxygen and can consume trace amounts of oxygen at bioremediation sites, which rapidly promotes the in situ biological system to achieve reducing conditions and lowers ORP [65]. The low ORP generated by nZVI in the subsurface environment may promote the respiration of *DHB* on organohalides by providing favorable growth conditions and shortening the dehalogenation lag period after biostimulation at bioremediation sites [67].

### 3.2. The Effects of Microorganisms on nZVI

Issues such as agglomeration and passivation limit the reactivity and mobility of nZVI. The interaction of microorganisms with nZVI is a convenient and environmentally friendly approach that can prevent passivation and enhance its reactivity. Microbial reduction helps maintain a low redox potential and consumes potential competing oxidants that may react with nZVI [63]. The microorganism *Shewanella oneidensis MR-1 (MR-1)* is widely distributed in aquatic environments; *MR-1* can utilize Fe (III) (hydr)oxides as electron acceptors, forming biofilms and inducing anaerobic respiration, which reduces Fe (III) to Fe (II). This process promotes the dissolution of the passivation layer on the nZVI surface and extends its reactivity; due to the widespread presence of MR-1 in natural water bodies, its use does not pose secondary pollution issues [68].

Other microorganisms also exhibit excellent Fe (III) reduction capabilities. For instance, *Penicillium oxalicum SL2 (P.oxalicum SL2)*, a promising nZVI-activating biomaterial, can dissolve iron precipitates deposited on the nZVI surface and regenerate them into Fe (II) [69]. *IRB* reduce Fe (III) to Fe (II) through co-metabolism, reversing the negative effects caused by corrosion products; this eliminates the electron transfer barriers between nZVI and target pollutants, reactivating the degradation activity of nZVI [57]. Microbial extracellular polymer substances (EPSs) can protect internal Fe^0^ from exposure to air, preventing the formation of a passivation layer on the nZVI surface. This reduces the concentration of hydroxyl radicals in the aquatic environment, increases the reactivity of nZVI, and enhances its reductive and adsorptive properties [70].

### 3.3. Mechanisms of Interaction between nZVI and Microorganisms

**Figure 4:**
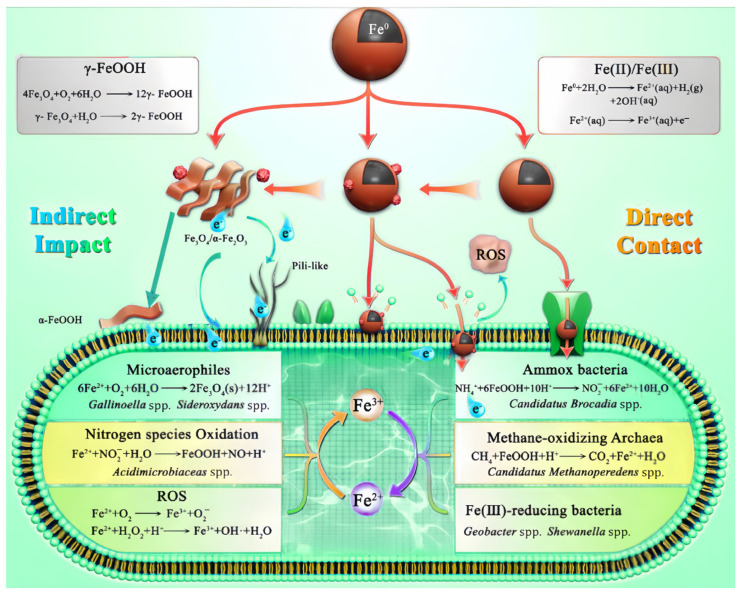
Interaction mechanisms between nZVI and microorganisms.

The interactions between microorganisms and nZVI can be divided into direct and indirect mechanisms. nZVI nanoparticles tend to adhere directly to the microbial surface due to electrostatic attraction, hydrogen bonding, or other driving forces; direct contact can influence microorganisms by enhancing direct electron transfer, providing iron nutrients, and generating reactive oxygen species (ROS), and indirect pathways mainly affect microorganisms by altering geochemical conditions or interacting with other elements. This effect could be realized through the formation of corrosion products or the interaction between iron and other elements [63]. The interaction pathways between nZVI and microorganisms are depicted in Figure 4 [63].

## 4. White Rot Fungi

**Figure 5:**
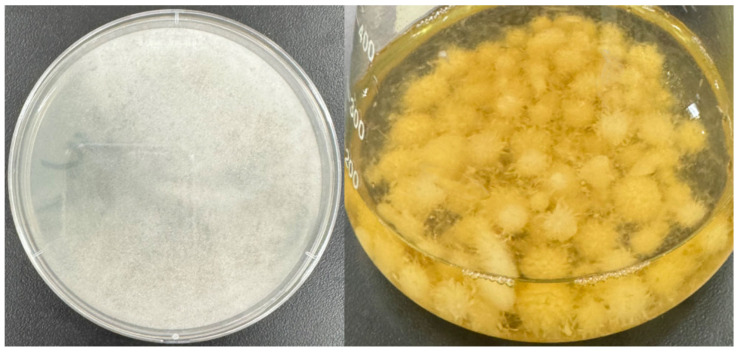
Morphology of the WRF *P. chrysosporium*.

WRF, classified under the class basidiomycetes, are microorganisms that cause white rot in wood or trees. Their hyphae can penetrate the cell cavities and release lignin-degrading enzymes, breaking down lignin into a white, spongy material [71]. Lignin-degrading enzymes are classified into two main categories: heme peroxidases and phenol oxidases [72]. Heme peroxidases consist of enzymes like lignin peroxidase (LiP), manganese peroxidase (MnP), and Mn (II): hydrogen peroxide oxidoreductase. Enzymes included in phenol oxidase are laccase (Lac) [73]. The lignin-degrading system of WRF plays a crucial role in the degradation of pollutants, especially LiP, which has a high redox potential (700–1400 mV); it can catalyze the degradation of various aromatic structures, such as veratryl alcohol (3,4-dimethoxybenzyl) and methoxybenzene [73]. MnP plays an important role in bioremediation owing to its significant potential for degradation. The morphology of the WRF *Phanerochaete chrysosporium (P. chrysosporium)* is shown in Figure 5.

### 4.1. Mechanisms of Pollutant Removal by White Rot Fungi

The removal and bioremediation of organic pollutants by WRF is a complex process that does not rely solely on one or a few enzymes. Instead, it involves the coordinated action of various components, ultimately leading to the efficient degradation of pollutants. The degradation of pollutants by WRF generally involves two stages: initially, various enzymes that can degrade pollutants are synthesized intracellularly, followed by their secretion into the extracellular space, where the degradation process occurs. WRF are capable of degrading and converting complex lignin macromolecules and xenobiotic compounds via the combined action of their enzyme systems and free radicals [2]. The enzyme system includes peroxidases that require H_2_O_2_ and oxidases that produce H_2_O_2_.

H_2_O_2_-dependent peroxidases are enzymes that use H_2_O_2_ to function and can oxidize and degrade lignin as well as lignin model compounds. The activity of peroxidases is critically dependent on H_2_O_2_ [74], such as LiP and MnP. Oxidase responsible for hydrogen peroxide production, including glucose oxidase (GOx), glyoxal oxidase (GLOX), and aryl-alcohol oxidase (AAO).

The removal of organic pollutants by LiP from WRF is presented in Table 1, while the removal of organic pollutants by MnP is presented in Table 2.

**Figure 6:**
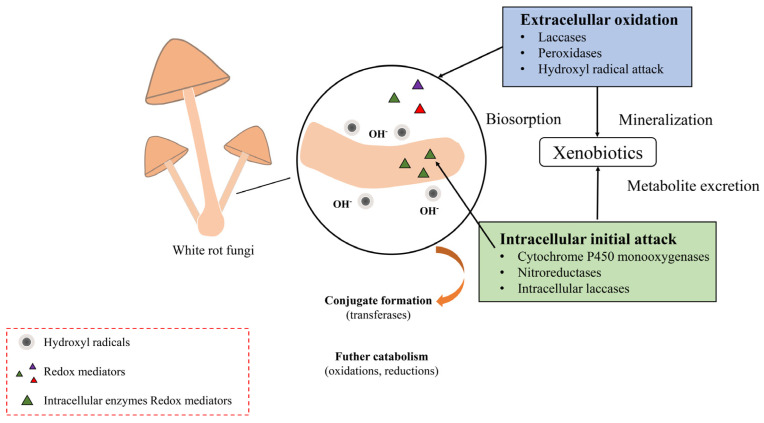
Primary mechanisms of xenobiotic degradation by WRF.

WRF produce extracellular enzymes such as LiP, MnP, and Lac. These enzymes are synthesized intracellularly and secreted extracellularly to perform their functions. Despite their non-selective nature, they can effectively degrade lignin and are thus commonly known as lignin-modifying enzymes (LMEs). The non-specific nature of these enzymes allows them to convert a wide range of persistent chemicals that have structures similar to lignin [92]. Because the chemical structures of many persistent pollutants resemble lignin, WRF demonstrate a significant ability to degrade a variety of pollutants through their unique metabolic types and mechanisms for extracellular macromolecule degradation. These pollutants include polycyclic aromatic hydrocarbons (PAHs) [93,94,95], polychlorinated biphenyls (PCBs) [96,97], pesticides [98,99], dyes [100,101], etc. Therefore, the degradation capability of WRF is not limited to lignin-like substances but also extends to other pollutants with similar chemical structures, providing a viable bioremediation approach for environmental pollutants. Figure 6 shows the mechanisms and enzymes associated with the initial intracellular attack and extracellular oxidation [102].

### 4.2. Remediation of Heavy Metal Pollutants by WRF

**Figure 7:**
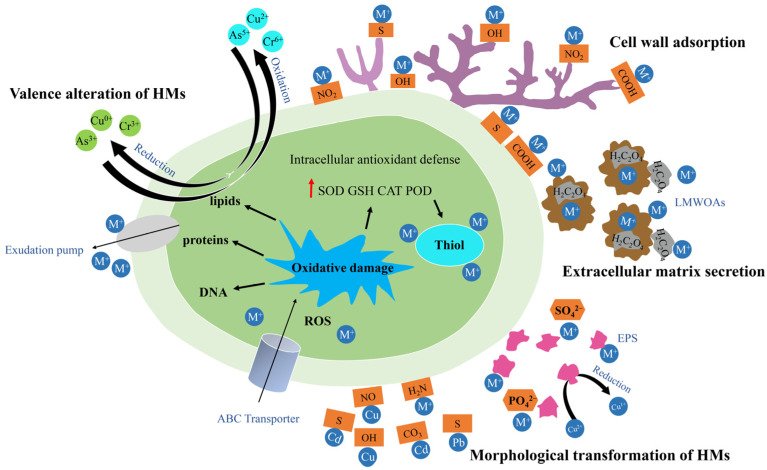
Mechanisms of WRF in the remediation of heavy metal pollutants.

WRF are effective not only in degrading organic pollutants but also in remediating heavy metal contamination. The primary mechanism for heavy metal removal by WRF is biosorption. The fungal cell walls possess various functional groups, including carboxyl, amino, and hydroxyl groups, which exhibit a high affinity for metal ions; these functional groups bind to heavy metal ions, thereby isolating and immobilizing them within the fungal biomass [103]. Lu et al. [104] developed a novel functionalized *P. chrysosporium* incorporating intracellular mineral scaffolds for the biosorption of heavy metal ions. According to the intraparticle diffusion model study, the biosorption process consists of three stages: an initial rapid surface adsorption stage, a slow transfer stage from the outer to the inner parts, and a final equilibrium stage. Sharma et al. [105] studied the bioremediation of industrial wastewater using the WRF *Phlebia brevispora* (*P. brevispora*) and *Phlebia floridensis* (*P. floridensis*) and compared their performance with *P. chrysosporium*. Spectroscopic analysis revealed that the maximum removal rates for nickel were 98–99%, for cadmium were 97–98%, and for lead were 12–98%. This indicates that WRF can serve as effective biosorbents for metal removal from industrial wastewater. Chen et al. [106] used viable *P. chrysosporium* mycelium to remove heavy metals Cd (II), Cu (II), and Zn (II) from aqueous solutions. The optimal biosorption conditions for the mycelium were found to be a pH of 5.5–6.5, a temperature of 37 °C, and an adsorption time of 6 h. Under these conditions, the fungal biosorbent achieved effective removal of Cd (II), Cu (II), and Zn (II), with maximum removal amounts of 59.77 mg/g, 74.78 mg/g, and 54.12 mg/g, respectively, demonstrating that *P. chrysosporium* is a promising biosorbent. *P. chrysosporium* was employed as a cost-effective and eco-friendly biosorbent for the simultaneous biosorption of Cd²⁺ and Ni²⁺. The biosorption process of *P. chrysosporium* is significantly influenced by factors such as pH, initial metal ion concentration, temperature, and contact time. Cd^²⁺^ and Ni^²⁺^ are biosorbed onto the surface of *P. chrysosporium* through interactions with functional groups on the mycelium that bind to the heavy metals. The cell wall of fungi plays a key role in the adsorption of heavy metals, with isolated cell wall fractions contributing 38–77% of the metal absorption; the adsorption capacity of these cell wall components is 20–50% greater than the total binding capacity of the mycelium [107,108,109]. In addition to biosorption, WRF can also achieve the degradation and removal of heavy metal pollutants by altering the speciation and oxidation states of heavy metals, producing oxidative stress responses and extracellular degradative enzymes, and activating intracellular antioxidant systems. Specifically, the transformation of heavy metal speciation includes the following: altering the composition of heavy metals through chemical precipitation, complexation, and crystallization processes to reduce the bioavailability of metal ions; changes in heavy metal oxidation states—intracellular redox systems such as flavin reductases, cytochromes, and hydrogenases reduce heavy metals to less toxic elemental forms by inducing electron donation; and intracellular antioxidant defenses—responding to oxidative stress by increasing levels of superoxide dismutase (SOD), catalase (CAT), peroxidase (POD), glutathione S-transferase (GST), and glutathione (GSH) [103,106]. The mechanisms of WRF in the remediation of heavy metal pollutants are illustrated in Figure 7 [106].

### 4.3. Limitations of WRF

Bioremediation is an environmentally friendly and sustainable method for pollutant removal; however, the application of WRF in biological treatment faces issues such as long degradation periods and low efficiency, and in practical applications, the degradation rate of pollutants may not meet the needs of short-term remediation. The long growth cycle of WRF, extended hydraulic retention time, and the low pH required for optimal enzyme activity make it challenging to maintain WRF in bioreactors [72]. Additionally, the degradation of organic pollutants by WRF is a part of secondary metabolism; since the concentration of micro-pollutants is insufficient to support the growth of WRF, they require additional carbon sources during their growth [110]. Additionally, the activity and stability of lignin-modifying enzymes secreted by WRF are also easily affected by factors such as temperature and pH.

## 5. nZVI Combined with WRF for Pollutant Remediation

**Figure 8:**
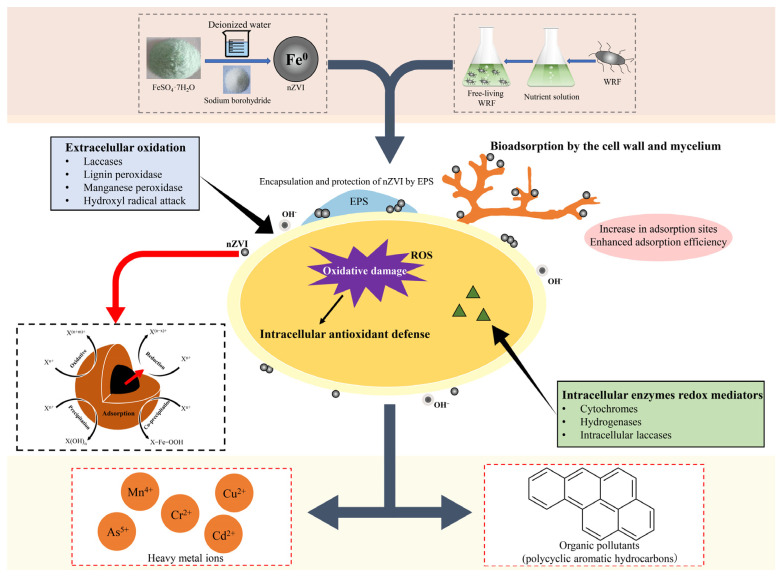
Conceptualization of pollutant remediation using a WRF-nZVI combined system.

nZVI and WRF have shown remarkable effectiveness in pollutant remediation. nZVI, with its superior specific surface area, reduction capability, and reactivity, exhibits excellent performance in the removal of heavy metals and organic pollutants, while WRF, with their powerful degradation ability, can efficiently degrade complex organic pollutants. However, research on the combined use of both for pollutant remediation is relatively scarce. Future studies could focus on exploring the mechanisms of the synergistic effects between nZVI and WRF and investigating their applications in different polluted environments. The conceptual model of pollutant remediation using the combined WRF and nZVI system is shown in Figure 8.

### 5.1. Enhanced Removal of Cd (II) from Water Using Combined WRF and nZVI

The study of cadmium removal using WRF faces issues of long processing time and low efficiency, while nZVI tends to aggregate and oxidize, reducing its reactivity, and also suffers from low cadmium removal efficiency and insufficient stability. Zeng et al. [111] employed a combined system of WRF and nZVI to enhance the removal of Cd (II) from water, investigating the effects of pH, initial Cd (II) concentration, temperature, and nZVI dosage on Cd (II) removal and analyzing the characteristics of nZVI’s impact on the intracellular and extracellular accumulation of cadmium by WRF. The results showed that under the conditions of pH = 6, initial Cd (II) concentration of 50 mg/L, temperature of 30 °C, and nZVI dosage of 0.1 g/L, the Cd (II) removal rate could reach more than 99.5% after 180 min of reaction.

WRF transport Cd (II) into the cells through ion channels on the cell wall and cell membrane, where it accumulates. The addition of nZVI may enhance the active transport capacity of WRF for Cd (II). The nZVI attached to the mycelium of WRF can increase the adsorption sites in the system, thereby improving the extracellular adsorption efficiency of WRF. Moreover, the WRF/nZVI system primarily removes Cd (II) through extracellular accumulation, and the addition of nZVI significantly increases the extracellular cadmium accumulation of WRF. Additionally, nZVI disrupts specific functional groups of fluorescent substances in EPS, causing the polymer backbone to break and bind with iron ions, promoting the formation of iron-containing minerals and enhancing Cd (II) removal. Furthermore, phosphate groups in WRF EPS can act as nucleation sites, fixing and aggregating Fe (II) and Fe (III) generated by nZVI oxidation, forming iron minerals rich in hydroxyl functional groups, such as ferrihydrite and magnetite, thus promoting the adsorption and fixation of Cd (II) in the solution.

### 5.2. The Combined Use of nZVI and White Rot Fungi Enhances the Degradation of PAHs

PAHs are common hazardous organic pollutants in soil. WRF can effectively degrade low molecular weight PAHs in liquid cultures, but their degradation efficiency for high molecular weight benzo[a]pyrene(Bap) is limited. The removal of Bap through bioremediation alone is relatively low. Tan [112] studied a comprehensive remediation method that combines WRF enhancement with persulfate oxidation activated by nZVI to improve the degradation of PAHs in soil. Direct application of nZVI/persulfate chemical oxidation can consume 55–75% of low molecular weight PAHs and 68% of Bap, while sequential treatment with nZVI/persulfate followed by inoculation with WRF can achieve a removal rate of 92–96% for all tested PAHs, significantly higher than a single treatment.

The increased PAH removal efficiency is attributed to the synergistic effects of chemical oxidation and microbial degradation. nZVI-activated persulfate generates sulfate radicals, which strongly oxidize high molecular weight PAHs, breaking them down into less toxic, lower molecular weight intermediates. These intermediates have higher bioavailability to WRF and are more easily further degraded. WRF utilize these oxidized intermediates, mineralizing them into carbon dioxide and water through a co-metabolic pathway. nZVI particles facilitate the oxidation and biodegradation of PAHs and metabolites by adsorbing them. The chemical oxidation process reduces the toxicity and molecular weight of PAHs, transforming them into more polar hydroxylated intermediates. These intermediates have higher bioavailability to WRF, improving fungal survival and activity. The overall synergistic effect greatly enhances the degradation efficiency, achieving more thorough PAH removal.

## 6. Conclusions

This paper reviews the application of nZVI and WRF in pollutant remediation. nZVI, as an efficient nanomaterial, has advantages such as a large specific surface area, strong reducibility, and high adsorption capacity. However, during use, it tends to agglomerate, oxidize, and passivate, which reduces its reactivity. To mitigate these issues, techniques such as sulfur modification (S-nZVI) and supported nZVI have achieved some success, enhancing the removal efficiency of nZVI for pollutants.

Although nZVI plays a crucial role in pollutant remediation, most contaminated sites still rely on bioremediation as a concurrent or final treatment approach to achieve remediation goals. Microbial communities are not only crucial for nutrient cycling in ecosystems, but they also play a key role in the degradation and immobilization of pollutants and heavy metals. The corrosion of nZVI reduces the redox potential, thereby creating an optimal environment for microbial growth. The hydrogen gas produced catalytically during nZVI corrosion acts as a preferred electron donor for numerous microorganisms. Moreover, the iron oxides and hydroxides formed during nZVI corrosion enhance microbial biodegradation efficiency by offering electron acceptors and decreasing pollutant toxicity. The iron precipitates assist in preventing the buildup of toxic metabolic byproducts, thereby further aiding in microbial survival. Additionally, nZVI does not adversely affect the overall abundance of microorganisms and, because of its high reactivity, can deplete trace oxygen at bioremediation sites. This swift achievement of reducing conditions in in-situ biological systems contributes to improved bioremediation efficiency. The process of microbial reduction is key in maintaining low oxidation–reduction potential (ORP) and in consuming potential competing oxidants that may react with nZVI. Certain microorganisms utilize Fe (III) (hydr)oxides as electron acceptors, leading to the formation of biofilms and the induction of anaerobic respiration, which reduces Fe (III) to Fe (II). This promotes the dissolution of the passivation layer on the surface of nZVI, thereby extending its reactive activity. Moreover, via co-metabolism, certain microorganisms are capable of reducing Fe (III) to Fe (II), counteracting the adverse effects of corrosion products and removing the electronic transfer barriers between nZVI and pollutants, thus reactivating the degradation potential of nZVI. In addition, microbial extracellular polymeric substances (EPSs) can shield the internal Fe0 from air contact, preventing the formation of a passivation layer on the nZVI surface. This process decreases the concentration of hydroxyl radicals in the aqueous environment, thereby increasing nZVI’s reactivity and enhancing its reductive and adsorptive capabilities, thereby facilitating pollutant degradation.

WRF, known for their distinctive metabolic pathways and extracellular degradation of macromolecules, demonstrate a strong ability to degrade various pollutants. The structural similarity of many recalcitrant pollutants to lignin enables these fungi to utilize their lignin-degrading enzyme system to break down a variety of organic contaminants. WRF have demonstrated good effectiveness in the biodegradation and adsorption of heavy metals, notably in the removal of metals like cadmium, nickel, and lead. Nonetheless, their use is somewhat constrained by issues such as long treatment durations and lower treatment efficiency.

Some studies have demonstrated that combining nZVI with WRF can overcome the limitations of both and enhance the efficiency of pollutant removal. In the context of heavy metal ion remediation, WRF actively transport and accumulate Cd (II) through ion channels located on the cell wall and membrane. The inclusion of nZVI enhances the fungi’s capacity for active Cd (II) transport and attaches to the fungal mycelium, thereby increasing the number of adsorption sites and improving extracellular adsorption efficiency. The WRF/nZVI composite system primarily removes Cd (II) via extracellular accumulation, with nZVI substantially boosting the fungi’s extracellular cadmium accumulation. Moreover, nZVI alters specific functional groups within extracellular polymeric substances (EPSs), allowing these groups to bind with iron ions and foster the formation of iron-containing minerals, thereby improving the removal of Cd (II). Phosphate groups within the extracellular polymeric substances (EPSs) of WRF act as nucleation sites, binding and accumulating Fe (II) and Fe (III) produced from nZVI oxidation. This process results in the formation of iron minerals abundant in hydroxyl functional groups, thereby facilitating the adsorption and stabilization of heavy metal ions. In addressing organic pollutants, nZVI can activate persulfate to produce sulfate radicals, which effectively oxidize high molecular weight organic pollutants such as PAHs, decomposing them into less toxic and smaller molecular weight intermediates. These intermediates are more bioavailable to WRF and can be further degraded more easily. The WRF then use these oxidized intermediates to mineralize them into carbon dioxide and water through co-metabolism. nZVI particles facilitate the oxidation and biodegradation of PAHs and their metabolites through adsorption. The chemical oxidation process decreases the toxicity and molecular weight of PAHs, converting them into more polar hydroxylated intermediates, which enhances their bioavailability to WRF and boosts fungal survival and activity. The combined effects significantly improve degradation efficiency, achieving more complete removal of PAHs.

The integration of nZVI with WRF effectively addresses their respective shortcomings and demonstrates significant synergistic effects. Nonetheless, there is currently limited research on the synergistic remediation of pollutants using WRF in conjunction with nZVI. Future studies should aim to explore and optimize their combined effects to enhance pollutant removal efficiency. This encompasses examining their interaction mechanisms under varying environmental conditions, identifying optimal parameters for synergistic systems, and evaluating the effectiveness of combined applications on various pollutants. A deeper understanding of the joint remediation potential of WRF and nZVI can lead to the development of more effective and sustainable pollution remediation technologies, fostering innovation and progress in environmental management and supporting sustainable development goals.

## Figures and Tables

**Table 1:** Removal of organic pollutants by LiP from WRF.

LiP Source	Pollutants	References
*P. chrysosporium* strain BKM F-1767 (DSM 6909)	Carbamazepine and diclofenac	[75]
*P. chrysosporium* strain BKM-F-1767	Tetracycline (TC) and oxytetracycline (OTC)	[76]
*P. chrysosporium*	Methylene blue (MB)	[77]
*P. chrysosporium*	Sulfides and carbonaceous matter in double refractory gold ore (DRGO)	[78]
*Phanerochaete sordida* YK-624 (*P. sordida* YK-624)	Five endocrine disruptors, p–t-octylphenol (OP), bisphenol A (BPA), estrone (E1), 17b-estradiol (E2), and ethinylestradiol (EE2)	[79]
*Lentinus squarrosulus* MPN12 (*L. squarrosulus* MPN12)	Synthetic dyes	[80]
The WRF *Irpex lacteus*	pyrene	[73]
*P. chrysosporium* ATCC 24725	Phenol, chlorophenol, and the dyes	[81]

**Table 2:** Removal of Organic Pollutants by MnP.

MnP Source	Pollutants and Concentration	References
*P. chrysosporium* strain BKM F-1767	Malachite green, 100 mg/L	[82]
*P. chrysosporium*	Textile wastewater	[83]
The WRF *Irpex lacteus*	PAHs (phenanthrene, anthracene, fluoranthene, and pyrene)	[84]
*P. chrysosporium* ME-446	The antimicrobial and preservative agent triclosan (TCS)	[85]
The WRF *Echinodontium taxodii* 2538 (*E. taxodii* 2538)	Nonphenolic and phenolic lignin model compounds	[86]
*P. chrysosporium* ME-446	The antifouling compound irgarol 1051	[87]
The White-rot *Basidiomycete, Pleurotus ostreatus*	2,2-Bis(4-hydroxyphenyl)propane (bisphenol A, BPA)	[88]
*Trametes* sp. 48424	Dyes (indigo, anthraquinone, azo, and triphenylmethane) and polycyclic aromatic hydrocarbons (PAHs) (fluorene, fluoranthene, pyrene, phenanthrene, and anthracene)	[89]
*P. chrysosporium*	Chlorinated phenolic compounds	[90]
*Trametes pubescens* i8	Synthetic dyes	[91]

## Data Availability

Not applicable.

## References

[1] Mansor M., Xu J. (2020). Benefits at the nanoscale: A review of nanoparticle-enabled processes favouring microbial growth and functionality. Environ. Microbiol..

[2] Munk L., Sitarz A.K., Kalyani D.C., Mikkelsen J.D., Meyer A.S. (2015). Can laccases catalyze bond cleavage in lignin?. Biotechnol. Adv..

[3] Huang D., Guo X., Peng Z., Zeng G., Xu P., Gong X., Deng R., Xue W., Wang R., Yi H. (2018). White rot fungi and advanced combined biotechnology with nanomaterials: Promising tools for endocrine-disrupting compounds biotransformation. Crit. Rev. Biotechnol..

[4] Zhou Q., Li J., Wang M., Zhao D. (2016). Iron-based magnetic nanomaterials and their environmental applications. Crit. Rev. Environ. Sci. Technol..

[5] Mukherjee R., Kumar R., Sinha A., Lama Y., Saha A.K. (2016). A review on synthesis, characterization, and applications of nano zero valent iron (nZVI) for environmental remediation. Crit. Rev. Environ. Sci. Technol..

[6] Ken D.S., Sinha A. (2020). Recent developments in surface modification of nano zero-valent iron (nZVI): Remediation, toxicity and environmental impacts. Environ. Nanotechnol. Monit. Manag..

[7] Glavee G.N., Klabunde K.J., Sorensen C.M., Hadjipanayis G.C. (1995). Chemistry of borohydride reduction of iron (II) and iron (III) ions in aqueous and nonaqueous media. Formation of nanoscale Fe, FeB, and Fe2B powders. Inorg. Chem..

[8] Qiu X.F.Z. (2010). Degradation of halogenated organic compounds by modified nano zero-valent iron. Prog. Chem..

[9] Li J., Zhang X., Sun Y., Liang L., Pan B., Zhang W., Guan X. (2017). Advances in sulfidation of zerovalent iron for water decontamination. Environ. Sci. Technol..

[10] Zhang W.-x. (2003). Nanoscale iron particles for environmental remediation: An overview. J. Nanoparticle Res..

[11] Dong H., He Q., Zeng G., Tang L., Zhang C., Xie Y., Zeng Y., Zhao F., Wu Y. (2016). Chromate removal by surface-modified nanoscale zero-valent iron: Effect of different surface coatings and water chemistry. J. Colloid Interface Sci..

[12] Montesinos V.N., Quici N., Litter M.I. (2014). Visible light enhanced Cr (VI) removal from aqueous solution by nanoparticulated zerovalent iron. Catal. Commun..

[13] Ramos M.A., Yan W., Li X.-q., Koel B.E., Zhang W.-x. (2009). Simultaneous oxidation and reduction of arsenic by zero-valent iron nanoparticles: Understanding the significance of the core−shell structure. J. Phys. Chem. C.

[14] Wang P., Wang Y., Liu T. (2021). Research progress of preparation of nano zero-valent iron by ball milling. Environ. Chem..

[15] Mu Y., Jia F., Ai Z., Zhang L. (2017). Iron oxide shell mediated environmental remediation properties of nano zero-valent iron. Environ. Sci. Nano.

[16] Zhu F., Ma S., Liu T., Deng X. (2018). Green synthesis of nano zero-valent iron/Cu by green tea to remove hexavalent chromium from groundwater. J. Clean. Prod..

[17] Fazlzadeh M., Rahmani K., Zarei A., Abdoallahzadeh H., Nasiri F., Khosravi R. (2017). A novel green synthesis of zero valent iron nanoparticles (NZVI) using three plant extracts and their efficient application for removal of Cr (VI) from aqueous solutions. Adv. Powder Technol..

[18] Hamzezadeh A., Fazlzadeh M., Rahmani K., Poureshgh Y. (2023). A novel green synthesis of zero valent iron nanoparticles (nZVI) using walnut green skin: Characterisation, catalytic degradation and toxicity studies. Int. J. Environ. Anal. Chem..

[19] Rashtbari Y., Hazrati S., Azari A., Afshin S., Fazlzadeh M., Vosoughi M. (2020). A novel, eco-friendly and green synthesis of PPAC-ZnO and PPAC-nZVI nanocomposite using pomegranate peel: Cephalexin adsorption experiments, mechanisms, isotherms and kinetics. Adv. Powder Technol..

[20] Wang T., Lin J., Chen Z., Megharaj M., Naidu R. (2014). Green synthesized iron nanoparticles by green tea and eucalyptus leaves extracts used for removal of nitrate in aqueous solution. J. Clean. Prod..

[21] Sun X., Kurokawa T., Suzuki M., Takagi M., Kawase Y. (2015). Removal of cationic dye methylene blue by zero-valent iron: Effects of pH and dissolved oxygen on removal mechanisms. J. Environ. Sci. Health Part A.

[22] Lu H.-J., Wang J.-K., Ferguson S., Wang T., Bao Y., Hao H.-x. (2016). Mechanism, synthesis and modification of nano zerovalent iron in water treatment. Nanoscale.

[23] Crane R.A., Scott T.B. (2012). Nanoscale zero-valent iron: Future prospects for an emerging water treatment technology. J. Hazard. Mater..

[24] de Lima Perini J.A., Silva B.F., Nogueira R.F.P. (2014). Zero-valent iron mediated degradation of ciprofloxacin–assessment of adsorption, operational parameters and degradation products. Chemosphere.

[25] Matheson L.J., Tratnyek P.G. (1994). Reductive dehalogenation of chlorinated methanes by iron metal. Environ. Sci. Technol..

[26] Zeng G., Liang D., Fan X., He Y., Zhang R., Lei X., Wei H., Sun D. (2024). Activated carbon fiber loaded nano zero-valent iron for Microcystis aeruginosa removal: Performance and mechanisms. Bioresour. Technol..

[27] Xiong M., Sun Y., Chai B., Fan G., Song G. (2023). Efficient peroxymonosulfate activation by magnetic CoFe_2_O_4_ nanoparticle immobilized on biochar toward sulfamethoxazole degradation: Performance, mechanism and pathway. Appl. Surf. Sci..

[28] Fu F., Dionysiou D.D., Liu H. (2014). The use of zero-valent iron for groundwater remediation and wastewater treatment: A review. J. Hazard. Mater..

[29] Mueller N.C., Braun J., Bruns J., Černík M., Rissing P., Rickerby D., Nowack B. (2012). Application of nanoscale zero valent iron (NZVI) for groundwater remediation in Europe. Environ. Sci. Pollut. Res..

[30] Greenlee L.F., Torrey J.D., Amaro R.L., Shaw J.M. (2012). Kinetics of zero valent iron nanoparticle oxidation in oxygenated water. Environ. Sci. Technol..

[31] Shi D., Zhu G., Zhang X., Zhang X., Li X., Fan J. (2019). Ultra-small and recyclable zero-valent iron nanoclusters for rapid and highly efficient catalytic reduction of p-nitrophenol in water. Nanoscale.

[32] Stefaniuk M., Oleszczuk P., Ok Y.S. (2016). Review on nano zerovalent iron (nZVI): From synthesis to environmental applications. Chem. Eng. J..

[33] Huang Q., Gu T., Liu A., Liu J., Zhang W.-x. (2021). Probing pollutant reactions at the iron surface: A case study on selenite reactions with nanoscale zero-valent iron. Environ. Sci. Nano.

[34] Zhou L., Li Z., Yi Y., Tsang E.P., Fang Z. (2022). Increasing the electron selectivity of nanoscale zero-valent iron in environmental remediation: A review. J. Hazard. Mater..

[35] Yan W., Herzing A.A., Kiely C.J., Zhang W.-x. (2010). Nanoscale zero-valent iron (nZVI): Aspects of the core-shell structure and reactions with inorganic species in water. J. Contam. Hydrol..

[36] Tang J., Tang L., Feng H., Dong H., Zhang Y., Liu S., Zeng G. (2017). Research progress of aqueous pollutants removal by sulfidated nanoscale zero-valent iron. Acta Chim. Sin..

[37] Wang H., Cai S., Shan L., Zhuang M., Li N., Quan G., Yan J. (2019). Adsorptive and reductive removal of chlorophenol from wastewater by biomass-derived mesoporous carbon-supported sulfide nanoscale zerovalent iron. Nanomaterials.

[38] He F., Li Z., Shi S., Xu W., Sheng H., Gu Y., Jiang Y., Xi B. (2018). Dechlorination of excess trichloroethene by bimetallic and sulfidated nanoscale zero-valent iron. Environ. Sci. Technol..

[39] Zhu X., Le T.T., Du J., Xu T., Cui Y., Ling H., Kim S.H. (2021). Novel core-shell sulfidated nano-Fe (0) particles for chromate sequestration: Promoted electron transfer and Fe (II) production. Chemosphere.

[40] Wu D., Peng S., Yan K., Shao B., Feng Y., Zhang Y. (2018). Enhanced As (III) sequestration using sulfide-modified nano-scale zero-valent iron with a characteristic core–shell structure: Sulfidation and as distribution. ACS Sustain. Chem. Eng..

[41] Li D., Zhong Y., Wang H., Huang W. (2021). Remarkable promotion in particle dispersion and electron transfer capacity of sulfidated nano zerovalent iron by coating alginate polymer. Sci. Total Environ..

[42] Wu Y., Yang M., Hu S., Wang L., Yao H. (2014). Characteristics and mechanisms of 4A zeolite supported nanoparticulate zero-valent iron as Fenton-like catalyst to degrade methylene blue. Toxicol. Environ. Chem..

[43] Qu R., Zhang W., Liu N., Zhang Q., Liu Y., Li X., Wei Y., Feng L. (2018). Antioil Ag_3_PO_4_ nanoparticle/polydopamine/Al2O3 sandwich structure for complex wastewater treatment: Dynamic catalysis under natural light. ACS Sustain. Chem. Eng..

[44] Chen Z., Wang T., Jin X., Chen Z., Megharaj M., Naidu R. (2013). Multifunctional kaolinite-supported nanoscale zero-valent iron used for the adsorption and degradation of crystal violet in aqueous solution. J. Colloid Interface Sci..

[45] Xuanyi Z., Zhe L., Jiawei C. (2018). Study on Mobility of Chitosan Coated Fe/Ni Bimetal Nanoparticles and Their Reactivity for Trichloroethylene Degradation in Groundwater. Geoscience.

[46] Kustov L.M., Finashina E.D., Shuvalova E.V., Tkachenko O.P., Kirichenko O.A. (2011). Pd–Fe nanoparticles stabilized by chitosan derivatives for perchloroethene dechlorination. Environ. Int..

[47] Xue J., Wang Z., Yang B., Zhou H., Huang Y., Yin Y., Liu J., Huang K. (2018). Degradation of Methylene Blue by Nano-Zero Valent Iron Loaded Garlic Residue. Adv. Environ. Prot..

[48] Cheng Y., Tang W., Sun X. (2018). Research on the Treatment of Phosphorus Containing Wastewater by Porous Ceramic Supported nZVI. Water Pollut. Treat..

[49] Shi L.-n., Lin Y.-M., Zhang X., Chen Z.-l. (2011). Synthesis, characterization and kinetics of bentonite supported nZVI for the removal of Cr (VI) from aqueous solution. Chem. Eng. J..

[50] Gil-Díaz M., Alonso J., Rodríguez-Valdés E., Gallego J., Lobo M.C. (2017). Comparing different commercial zero valent iron nanoparticles to immobilize As and Hg in brownfield soil. Sci. Total Environ..

[51] Eljamal R., Eljamal O., Maamoun I., Yilmaz G., Sugihara Y. (2020). Enhancing the characteristics and reactivity of nZVI: Polymers effect and mechanisms. J. Mol. Liq..

[52] Mokete R., Eljamal O., Sugihara Y. (2020). Exploration of the reactivity of nanoscale zero-valent iron (NZVI) associated nanoparticles in diverse experimental conditions. Chem. Eng. Process.-Process Intensif..

[53] Xie Y., Dong H., Zeng G., Tang L., Jiang Z., Zhang C., Deng J., Zhang L., Zhang Y. (2017). The interactions between nanoscale zero-valent iron and microbes in the subsurface environment: A review. J. Hazard. Mater..

[54] Yao B., Luo Z., Zhi D., Hou D., Luo L., Du S., Zhou Y. (2021). Current progress in degradation and removal methods of polybrominated diphenyl ethers from water and soil: A review. J. Hazard. Mater..

[55] Ali H. (2010). Biodegradation of synthetic dyes—A review. Water Air Soil Pollut..

[56] Jabbar N.M., Alardhi S.M., Mohammed A.K., Salih I.K., Albayati T.M. (2022). Challenges in the implementation of bioremediation processes in petroleum-contaminated soils: A review. Environ. Nanotechnol. Monit. Manag..

[57] Dong H., Li L., Lu Y., Cheng Y., Wang Y., Ning Q., Wang B., Zhang L., Zeng G. (2019). Integration of nanoscale zero-valent iron and functional anaerobic bacteria for groundwater remediation: A review. Environ. Int..

[58] Huang M., Wang X., Zhao F. (2021). Research progress of zero-valent-iron microbial coupled system in remediating contaminated groundwater. China Environ. Sci..

[59] Reardon E.J., Fagan R., Vogan J.L., Przepiora A. (2008). Anaerobic corrosion reaction kinetics of nanosized iron. Environ. Sci. Technol..

[60] Liu Y., Lowry G.V. (2006). Effect of particle age (Fe^0^ content) and solution pH on NZVI reactivity: H_2_ evolution and TCE dechlorination. Environ. Sci. Technol..

[61] Haferburg G., Kothe E. (2007). Microbes and metals: Interactions in the environment. J. Basic Microbiol..

[62] Klueglein N., Zeitvogel F., Stierhof Y.-D., Floetenmeyer M., Konhauser K.O., Kappler A., Obst M. (2014). Potential role of nitrite for abiotic Fe (II) oxidation and cell encrustation during nitrate reduction by denitrifying bacteria. Appl. Environ. Microbiol..

[63] Liu N., Liu J., Wang H., Li S., Zhang W.-x. (2022). Microbes team with nanoscale zero-valent iron: A robust route for degradation of recalcitrant pollutants. J. Environ. Sci..

[64] Wu D., Shen Y., Ding A., Mahmood Q., Liu S., Tu Q. (2013). Effects of nanoscale zero-valent iron particles on biological nitrogen and phosphorus removal and microorganisms in activated sludge. J. Hazard. Mater..

[65] Kirschling T.L., Gregory K.B., Minkley J., Edwin G., Lowry G.V., Tilton R.D. (2010). Impact of nanoscale zero valent iron on geochemistry and microbial populations in trichloroethylene contaminated aquifer materials. Environ. Sci. Technol..

[66] Niu C., Cai T., Lu X., Zhen G., Pan Y., Ren X., Qin X., Li W., Tang Y., Zhi Z. (2021). Nano zero-valent iron regulates the enrichment of organics-degrading and hydrogenotrophic microbes to stimulate methane bioconversion of waste activated sludge. Chem. Eng. J..

[67] Wang S., Chen S., Wang Y., Low A., Lu Q., Qiu R. (2016). Integration of organohalide-respiring bacteria and nanoscale zero-valent iron (Bio-nZVI-RD): A perfect marriage for the remediation of organohalide pollutants?. Biotechnol. Adv..

[68] Ma L., Du Y., Chen S., Zhang F., Zhan W., Du D., Zhang T.C. (2021). Nanoscale zero-valent iron coupling with Shewanella oneidensis MR-1 for enhanced reduction/removal of aqueous Cr (VI). Sep. Purif. Technol..

[69] Luo Y., Pang J., Peng C., Ye J., Long B., Tong J., Shi J. (2023). Cr (VI) Reduction and Fe (II) Regeneration by Penicillium oxalicum SL2-Enhanced Nanoscale Zero-Valent Iron. Environ. Sci. Technol..

[70] Zhou L., Li A., Ma F., Zhao H., Deng F., Pi S., Tang A., Yang J. (2020). Combining high electron transfer efficiency and oxidation resistance in nZVI with coatings of microbial extracellular polymeric substances to enhance Sb (V) reduction and adsorption. Chem. Eng. J..

[71] Gao D., Du L., Yang J., Wu W.-M., Liang H. (2010). A critical review of the application of white rot fungus to environmental pollution control. Crit. Rev. Biotechnol..

[72] Rodríguez-Couto S. (2017). Industrial and environmental applications of white-rot fungi. Mycosphere.

[73] Suryadi H., Judono J.J., Putri M.R., Eclessia A.D., Ulhaq J.M., Agustina D.N., Sumiati T. (2022). Biodelignification of lignocellulose using ligninolytic enzymes from white-rot fungi. Heliyon.

[74] Kotterman M., Wasseveld R.A., Field J.A. (1996). Hydrogen peroxide production as a limiting factor in xenobiotic compound oxidation by nitrogen-sufficient cultures of Bjerkandera sp. strain BOS55 overproducing peroxidases. Appl. Environ. Microbiol..

[75] Zhang Y., Geißen S.-U. (2010). In vitro degradation of carbamazepine and diclofenac by crude lignin peroxidase. J. Hazard. Mater..

[76] Wen X., Jia Y., Li J. (2009). Degradation of tetracycline and oxytetracycline by crude lignin peroxidase prepared from *Phanerochaete chrysosporium*–A white rot fungus. Chemosphere.

[77] Ferreira-Leitao V.S., de Carvalho M.E.A., Bon E.P. (2007). Lignin peroxidase efficiency for methylene blue decolouration: Comparison to reported methods. Dye. Pigment..

[78] Konadu K.T., Harrison S.T., Osseo-Asare K., Sasaki K. (2019). Transformation of the carbonaceous matter in double refractory gold ore by crude lignin peroxidase released from the white-rot fungus. Int. Biodeterior. Biodegrad..

[79] Wang J., Majima N., Hirai H., Kawagishi H. (2012). Effective removal of endocrine-disrupting compounds by lignin peroxidase from the white-rot fungus *Phanerochaete sordida* YK-624. Curr. Microbiol..

[80] Dinh Giap V., Nghi D.H., Cuong L.H., Quynh D.T. (2022). Lignin Peroxidase from the White-rot Fungus *Lentinus squarrosulus* MPN12 and its Application in the Biodegradation of Synthetic Dyes and Lignin. BioResources.

[81] Manimekalai R., Swaminathan T. (2000). Removal of hazardous compounds by lignin peroxidase from *Phanerochaete chrysosporium*. Bioprocess Eng..

[82] Saravanakumar T., Palvannan T., Kim D.-H., Park S.-M. (2013). Manganese peroxidase H4 isozyme mediated degradation and detoxification of triarylmethane dye malachite green: Optimization of decolorization by response surface methodology. Appl. Biochem. Biotechnol..

[83] Bilal M., Asgher M., Iqbal M., Hu H., Zhang X. (2016). Chitosan beads immobilized manganese peroxidase catalytic potential for detoxification and decolorization of textile effluent. Int. J. Biol. Macromol..

[84] Baborová P., Möder M., Baldrian P., Cajthamlová K., Cajthaml T. (2006). Purification of a new manganese peroxidase of the white-rot fungus *Irpex lacteus*, and degradation of polycyclic aromatic hydrocarbons by the enzyme. Res. Microbiol..

[85] Inoue Y., Hata T., Kawai S., Okamura H., Nishida T. (2010). Elimination and detoxification of triclosan by manganese peroxidase from white rot fungus. J. Hazard. Mater..

[86] Kong W., Chen H., Lyu S., Ma F., Yu H., Zhang X. (2016). Characterization of a novel manganese peroxidase from white-rot fungus *Echinodontium taxodii* 2538, and its use for the degradation of lignin-related compounds. Process Biochem..

[87] Ogawa N., Okamura H., Hirai H., Nishida T. (2004). Degradation of the antifouling compound Irgarol 1051 by manganese peroxidase from the white rot fungus *Phanerochaete chrysosporium*. Chemosphere.

[88] Hirano T., HoNDA Y., Watanabe T., KUwAHARA M. (2000). Degradation of bisphenol A by the lignin-degrading enzyme, manganese peroxidase, produced by the white-rot basidiomycete, *Pleurotus ostreatus*. Biosci. Biotechnol. Biochem..

[89] Zhang H., Zhang S., He F., Qin X., Zhang X., Yang Y. (2016). Characterization of a manganese peroxidase from white-rot fungus *Trametes* sp. 48424 with strong ability of degrading different types of dyes and polycyclic aromatic hydrocarbons. J. Hazard. Mater..

[90] Rubilar O., Diez M.C., Gianfreda L. (2008). Transformation of chlorinated phenolic compounds by white rot fungi. Crit. Rev. Environ. Sci. Technol..

[91] Rekik H., Jaouadi N.Z., Bouacem K., Zenati B., Kourdali S., Badis A., Annane R., Bouanane-Darenfed A., Bejar S., Jaouadi B. (2019). Physical and enzymatic properties of a new manganese peroxidase from the white-rot fungus *Trametes pubescens* strain i8 for lignin biodegradation and textile-dyes biodecolorization. Int. J. Biol. Macromol..

[92] Mansur M., Arias M.a.E., Copa-Patino J.L., Flärdh M.a., González A.E. (2003). The white-rot fungus *Pleurotus ostreatus* secretes laccase isozymes with different substrate specificities. Mycologia.

[93] Chane A.D., Košnář Z., Hřebečková T., Jozífek M., Doležal P., Tlustoš P. (2024). Persistent polycyclic aromatic hydrocarbons removal from sewage sludge-amended soil through phytoremediation combined with solid-state ligninolytic fungal cultures. Fungal Biol..

[94] Lee H., Jang Y., Choi Y.-S., Kim M.-J., Lee J., Lee H., Hong J.-H., Lee Y.M., Kim G.-H., Kim J.-J. (2014). Biotechnological procedures to select white rot fungi for the degradation of PAHs. J. Microbiol. Methods.

[95] Kadri T., Rouissi T., Brar S.K., Cledon M., Sarma S., Verma M. (2017). Biodegradation of polycyclic aromatic hydrocarbons (PAHs) by fungal enzymes: A review. J. Environ. Sci..

[96] Novotný Č., Vyas B., Erbanova P., Kubatova A., Šašek V. (1997). Removal of PCBs by various white rot fungi in liquid cultures. Folia Microbiol..

[97] Stella T., Covino S., Čvančarová M., Filipová A., Petruccioli M., D’Annibale A., Cajthaml T. (2017). Bioremediation of long-term PCB-contaminated soil by white-rot fungi. J. Hazard. Mater..

[98] Gouma S., Papadaki A.A., Markakis G., Magan N., Goumas D. (2019). Studies on pesticides mixture degradation by white rot fungi. J. Ecol. Eng..

[99] Xin W., Zhaoxing L., Zijun N., Lei S. (2020). Research progress on degradation of pesticides by white rot fungi. Chin. J. Pestic. Sci..

[100] Wesenberg D., Kyriakides I., Agathos S.N. (2003). White-rot fungi and their enzymes for the treatment of industrial dye effluents. Biotechnol. Adv..

[101] Sen S.K., Raut S., Bandyopadhyay P., Raut S. (2016). Fungal decolouration and degradation of azo dyes: A review. Fungal Biol. Rev..

[102] Torres-Farradá G., Thijs S., Rineau F., Guerra G., Vangronsveld J. (2024). White Rot Fungi as Tools for the Bioremediation of Xenobiotics: A Review. J. Fungi.

[103] Latif W., Ciniglia C., Iovinella M., Shafiq M., Papa S. (2023). Role of White Rot Fungi in Industrial Wastewater Treatment: A Review. Appl. Sci..

[104] Lu N., Hu T., Zhai Y., Qin H., Aliyeva J., Zhang H. (2020). Fungal cell with artificial metal container for heavy metals biosorption: Equilibrium, kinetics study and mechanisms analysis. Environ. Res..

[105] Sharma K., Giri R., Sharma R. (2023). Efficient bioremediation of metal containing industrial wastewater using white rot fungi. Int. J. Environ. Sci. Technol..

[106] Chen L., Zhang X., Zhang M., Zhu Y., Zhuo R. (2022). Removal of heavy-metal pollutants by white rot fungi: Mechanisms, achievements, and perspectives. J. Clean. Prod..

[107] Gabriel J., Baldrian P., Hladíková K., Háková M. (2001). Copper sorption by native and modified pellets of wood-rotting basidiomycetes. Lett. Appl. Microbiol..

[108] Yetis U., Dolek A., Dilek F.B., Ozcengiz G. (2000). The removal of Pb (II) by *Phanerochaete chrysosporium*. Water Res..

[109] Baldrian P. (2003). Interactions of heavy metals with white-rot fungi. Enzym. Microb. Technol..

[110] Mir-Tutusaus J.A., Baccar R., Caminal G., Sarrà M. (2018). Can white-rot fungi be a real wastewater treatment alternative for organic micropollutants removal? A review. Water Res..

[111] Zeng Q., Zhou X., Huang C., Wang P., Cheng H., Zheng X., Wang M., Liu H. (2022). Enhanced removal of Cd(II) from aqueous solution by nanoscale zero-valent iron coupled with white rot fungus. China Environ. Sci..

[112] Tan X. (2023). Remediation of PAHs contaminated soil enhanced by nano-zero-valent iron combined with white rot fungi *Peniophora incarnata*. Alex. Eng. J..

